# Education for corrections officers to better meet the mental health needs of inmates

**DOI:** 10.1192/bjo.2021.379

**Published:** 2021-06-18

**Authors:** Shaheen Darani, Kiran Patel, Laura Hayos, Tanya Connors, Faisal Islam, Anika Saiva, Sandy Simpson

**Affiliations:** 1 University of Toronto and Centre for Addiction and Mental Health; 2 Centre for Faculty Development, University of Toronto; 3 Centre for Addiction and Mental Health; 4 HBSc, MPH, Centre for Addiction and Mental Health

## Abstract

**Aims:**

In Canada, there has been an increase in the rate of incarceration of individuals with mental health diagnoses. Overrepresentation of individuals with psychiatric diagnoses in correctional settings is well-established. Front-line officers play a central role in dealing with mental health struggles of inmates. Nonetheless, the training that officers receive is often considered inadequate. To address this gap, the goal of this study was to design, implement, and evaluate a mental health training for correctional officers at the Toronto South Detention Centre (TSDC) and Vanier Centre for Women (VCW) in Ontario, Canada.

**Method:**

A needs assessment was undertaken among officers at the TSDC. In response to needs identified, a one-day course was delivered to officers (n = 57) at the TSDC and VCW (n = 41). The curriculum included mental health awareness; assessment of risk; communicating with inmates in distress; and self-care. Live simulations provided the opportunity for participants to identify signs of mental illness, assess risk, and respond strategically to de-escalate situations. Participants’ knowledge and confidence in their ability to identify and assist individuals with these problems was established using pre and post measures. Participant satisfaction was also measured via a survey. A three-month follow-up administration was used to determine maintenance of gains. Focus groups at nine months were conducted to understand participants’ needs, learning, and impact of training.

**Result:**

The results were promising, with 92% and 88% of participants at TSDC and Vanier Centre for Women respectively expressing satisfaction and 62% and 68% at TSDC and Vanier Centre for Women respectively stating they intended to change practices. Analyses of change in knowledge and confidence scores pre to post-training showed statistically significant improvement in all areas measured. Three-month follow-up at TSDC showed 75% of respondents have applied what they learned from the training to a “moderate or great extent”. Focus group themes showed improved attitudes and ability to identify behaviours related to inmate mental health struggles and interest in further training to support officers’ mental health.

**Conclusion:**

This study shows that training informed by officer learning needs can help them better meet the mental health needs of inmates. Training can improve attitudes toward inmates presenting with mental health issues. Training that is interactive and provides skills practice can have sustained impact on practice. Further training should integrate self-care to support officers' mental health.

